# Ultra-fast fabrication of Bi_2_Te_3_ based thermoelectric materials by flash-sintering at room temperature combining with spark plasma sintering

**DOI:** 10.1038/s41598-022-14405-5

**Published:** 2022-06-16

**Authors:** Zhiwei Zhang, Minna Sun, Jinchao Liu, Lili Cao, Mengran Su, Qingwei Liao, Yuan Deng, Lei Qin

**Affiliations:** 1 Beijing Key Laboratory for Sensors, Beijing Information Science & Technology University, Beijing, 100192 China; 2 Beijing Key Laboratory for Optoelectronic Measurement Technology, Beijing Information Science & Technology University, Beijing, 100192 China; 3 AECC Aero Engine Academy of China, Beijing, 101304 China; 4 Research Institute for Frontier Science, Beihang University, Beijing, 100083 China

**Keywords:** Materials for energy and catalysis, Energy harvesting

## Abstract

Highly crystalline Bi_2_Te_3_ based compounds with small grain size were successfully synthesized by flash sintering (FS) method in 10 s at room temperature under suitable current density using Bi, Te and Se powders. The instantaneously generated local Joule heat at grain boundary is regarded as the main reason for the rapid completion of chemical reaction and crystallization. By combining FS synthesis method with spark plasma sintering (SPS), Bi_2_Te_3_ based bulk materials with high relative density were fabricated in 10 min. Suitably prolonging sintering temperature and holding time in SPS process can decrease carrier concentration and phonon thermal conductivity, while increasing carrier mobility. Hence, the sample prepared at 753 K for 3 min shows 20% higher *ZT* value than that of the sample prepared at 723 K for 3 min. Compared with common zone melting or powder metallurgy methods taking several hours by complex operation, this method is time-saving and low cost.

## Introduction

With the increasingly serious energy and environmental crisis, exploring clean energy resources and green energy conversion technologies become urgent and mandatory for the researchers in related fields^1^. Thermoelectric material is a kind of functional material which can realize the direct conversion between heat and electricity by Seebeck effect and Peltier effect^2^. Because of its pure solid state operation mode, high stability and long life, thermoelectric technology provides opportunities to harvest useful electricity from waste heat or to realize in situ refrigeration^3–5^. The conversion performance of thermoelectric material is usually evaluated by the dimensionless figure of merit *ZT*, which can be expressed as *ZT* = *S*^2^*σT*/*κ*, where *S* is the Seebeck coefficient, *σ* is the electrical conductivity, *κ* is the thermal conductivity (including electronic thermal conductivity *κ*_e_, lattice thermal conductivity *κ*_l_ and bipolar thermal conductivity *κ*_b_) and *T* is the absolute temperature^6^. Therefore, high electrical conductivity, large Seebeck coefficient and low thermal conductivity should be simultaneously owned for an ideal thermoelectric material^7^. Bismuth telluride (Bi_2_Te_3_) is a kind of narrow band gap semiconductor material, which has relatively high electrical conductivity, large Seebeck coefficient and low thermal conductivity between 300–500 K^8^. Up to now, bismuth telluride and its alloys with antimony telluride or bismuth selenide have been the most mature thermoelectric material systems to be used at near room temperature^9,10^. Some commercial thermoelectric devices and modules for power generation and solid-state cooling at this temperature range have been successfully developed based on bismuth telluride and its alloys^11^.

Nowadays, commercial bismuth telluride based thermoelectric materials are generally produced by zone melting (ZM) method. Although ZM technique is convenient, its fabrication process is rather time consuming and energy intensive^12^. The prolonged annealing at high temperature may also lead to composition deviation, thus deteriorating thermoelectric performance. Moreover, the fabricated products are highly oriented and can cleave easily along the basal plane, thereby resulting in poor mechanical properties and limiting long-term usage^13^. Therefore, powder metallurgy techniques combining with advanced sintering techniques were explored extensively in recent years to fabricate polycrystalline bismuth telluride based thermoelectric materials with both high mechanical and thermoelectric properties. For example, high energy ball milling, melt spinning and various wet chemical methods have been successfully developed to synthesize Bi_2_Te_3_ based compounds with small grain size and modulated microstructure^14–17^. By combining these synthesis methods with hot pressing (HP) or spark plasma sintering (SPS) techniques, relatively robust mechanical properties and high thermoelectric performance have been simultaneously achieved in bismuth telluride based bulk materials. However, these synthesis methods are still time consuming and energy intensive, and the products may be oxidized or polluted by medium and organic impurities in the synthetic process, resulting in deteriorated thermoelectric performance^18^. Therefore, it is of great interest to develop new fabrication methods for high performance polycrystalline bismuth telluride based thermoelectric materials with low cost, high efficiency and simple craft process.

Over the past 10 years, flash sintering (FS) has been demonstrated as a novel technique to fabricate ceramic materials. Under the combined action of external electric field and thermal field, the sintering densification of ceramics can be achieved at low temperature within a few seconds^19–21^. Therefore, flash sintering is much more cost-effective and energy-efficient than traditional high temperature sintering method taking several hours. Recently, this technique has been successfully extended to reactive FS, which can induce both phase transformation and sintering densification. For example, dense Na_0.5_K_0.5_NbO_3_ ceramics have been reported to be fabricated with NaNbO_3_ and KNbO_3_ mixed powders by reactive FS, in which the chemical reaction and sintering occur simultaneously^22^. This rapid preparation method at low temperature can effectively reduce the loss of volatile elements and restrain grain growth, which are beneficial for the enhancement of thermoelectric performance. Therefore, flash sintering shows tremendous potential in the fabrication of high-performance thermoelectric materials. However, up to now there are few papers related to the FS of thermoelectric materials. The preparations of thermoelectric materials are generally realized by a flash derivative that is called flash-SPS, which uses SPS equipment to produce rapid heating rate similar to FS^23–25^. In the process of flash-SPS, no mold is used so that current passes directly through the cold pressed or pre-densified sample held directly between graphite punches. Actually, flash-SPS is used as a rapid sintering method for the densification of bulk thermoelectric materials with pre-synthesized compounds as raw materials^26–29^. For example, the fabrications of highly dense Sb_2_Te_3_ bulk materials were conducted by flash-SPS with a current feed duration of 1 s using commercial Sb_2_Te_3_ raw powder^29^. However, nearly no literature has been reported for the synthesis of thermoelectric compounds by FS.

In this work, we demonstrate that Bi_2_Te_3_ based compounds can be synthesized efficiently by FS method through adjusting suitable current density. The results indicate that highly crystalline Bi_2_Te_3_ based compounds with small grain size can be synthesized in 10 s at room temperature using commercial Bi, Te and Se powders with much larger size by an ordinary direct current power supply. By combining FS with SPS, Bi_2_Te_3_ based bulk materials with nominal composition and high density can be prepared. Hence, this method is hopeful for the mass production of bismuth telluride based thermoelectric materials.

## Results and discussion

### Phase composition and microstructure

**Figure 1:**
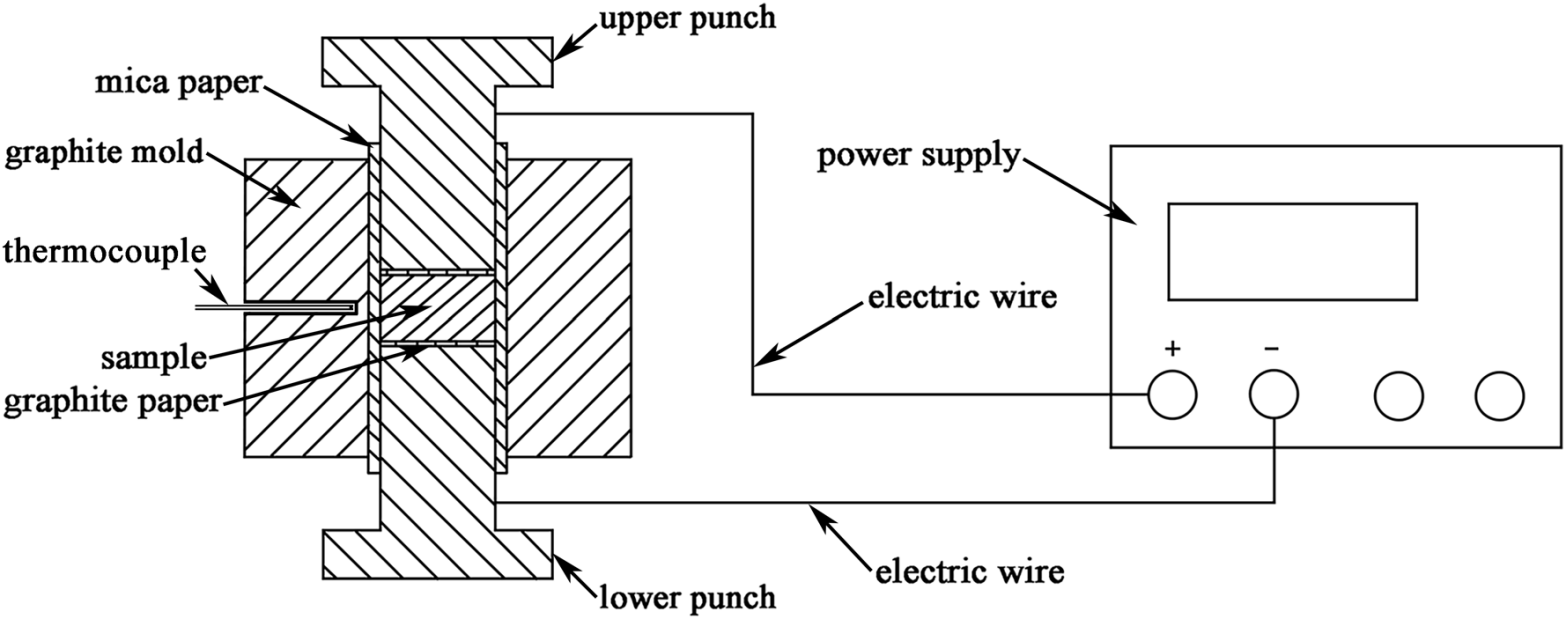
Schematic diagram of the FS apparatus.

Schematic diagram of the FS apparatus is shown in Fig. 1. The mixture of Bi and Te raw powders according to the stoichiometric composition of Bi_2_Te_3_ was loaded into a graphite mold, the inner wall of which was insulated by a piece of mica paper to ensure that current only passed through the mixture rather than the graphite mold. A thermocouple was inserted into a Φ1.8 mm hole in the middle of the graphite mold to measure the transient surface temperature of the sample. Two pieces of graphite papers were loaded between the sample and the punches for easily taking out of the product. The mixed powder was compacted by the upper and lower punches on a tablet press, and then the pressure was released. Whereafter, direct current with certain density passed through the compacted mixture to realize the FS of Bi_2_Te_3_ compound. Generally, tellurium is partially substituted by selenium to optimize the carrier and thermal transport properties of Bi_2_Te_3_. Hence, another sample with the stoichiometric composition of Bi_2_Te_2.7_Se_0.3_ was also synthesized using Bi, Te and Se powders by FS. The synthesized Bi_2_Te_3−x_Se_x_ compounds by FS were then ground to powders for SPS.

**Figure 2:**
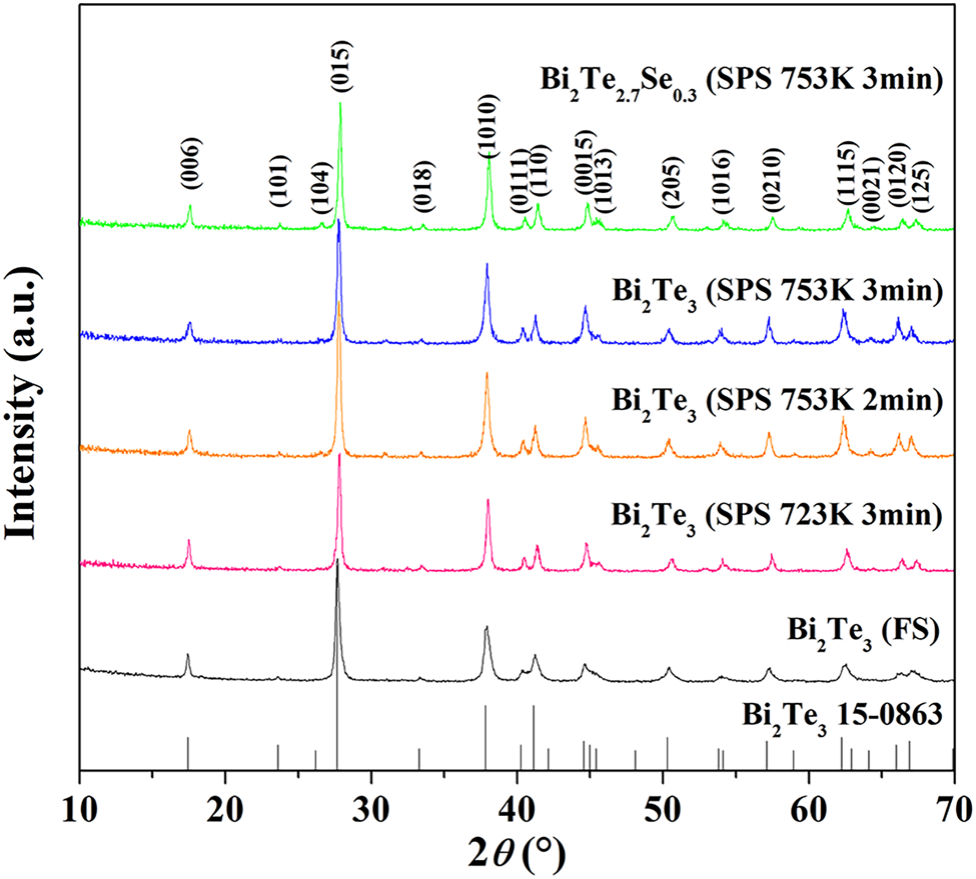
XRD patterns of the Bi_2_Te_3_ compound synthesized by FS and the Bi_2_Te_3−x_Se_x_ bulk samples further prepared by SPS on different conditions.

XRD patterns of the Bi_2_Te_3_ compound synthesized by FS and the Bi_2_Te_3−x_Se_x_ bulk samples further prepared by SPS on different conditions are shown in Fig. 2. For the Bi_2_Te_3_ compound synthesized by FS, all diffraction peaks are well indexed to the standard JCPDS card (PDF#15-0863) of Bi_2_Te_3_ with rhombohedral crystal structure and no peak related to oxidation or impurity can be detected, indicating that single phase Bi_2_Te_3_ compound with high crystallinity can be synthesized by FS in 10 s at room temperature using Bi and Te powders. As illustrated in Table. 1, the compound synthesized by FS has well matched composition to the desired stoichiometry of Bi_2_Te_3_ as measured by ICPOES, indicating that no volatilization of Te occurred in the process of FS. The transient surface temperature of the sample synthesized by FS was measured to be 625 K when current passed through the sample for 1 s, meaning that an order of 10^4^ K/min self-heating rate of the specimen had been achieved. This extremely rapid Joule heating occurring on the macroscale is considered to be attributed to the heat localization at grain boundary, which may result in the instantaneous occurrences of chemical reaction and crystallization^30^. In the experiment, we found that there is a threshold value of current density for the synthesis of Bi_2_Te_3_ compound by FS, which is about 8 A/cm^2^. Below the current density of 8 A/cm^2^, FS does not happen because of the deficient Joule heat. Furthermore, volume expansion of the sample was observed after FS. Considering the transient surface temperature of the sample in FS process is much higher than the melting point of Bi (544.6 K), the synthetic process should include the melting of Bi and subsequent chemical reaction with Te to form Bi_2_Te_3_ based compounds. Because of the volume expansion after FS, the relative density of the product is only about 70.4% calculated by the theoretical density 7.73 g cm^−3^. The Bi_2_Te_3_ based compounds synthesized by FS were then ground to powder and sintered further to compact pellets by SPS on different conditions. The main peaks of the Bi_2_Te_3−x_Se_x_ bulk samples further prepared by SPS on different conditions are also well in accordance with the standard JCPDS card (PDF#15-0863) of Bi_2_Te_3_. After SPS, the samples show higher relative intensity of (00* l*) peaks, indicating the enhanced preferential orientation of basal (00* l*) planes perpendicular to the pressing direction. To investigate the degree of preferred orientation of (00* l*) planes, orientation factors *F* were calculated using Lotgering method, which can be expressed by the following formulae:

1 $$F = \frac{{P - P_{0} }}{{1 - P_{0} }}$$

2 $$P = \frac{{I\left( {00l} \right)}}{{\sum {I\left( {hkl} \right)} }},\;P_{0} = \frac{{I_{0} \left( {00l} \right)}}{{\sum {I_{0} \left( {hkl} \right)} }}$$

where *I*(hkl) and *I*_0_(hkl) are the peak integral intensities for the measured and randomly oriented samples, respectively^31,32^. The orientation degree was enhanced with the increase of sintering temperature and holding time in SPS process, as indicated in Table. 1. However, the highest value of *F* obtained for the sample sintered at 753 K for 3 min is only about 0.095, which indicates that almost no highly oriented textures in the samples were formed in SPS process although bismuth telluride tends to grow along the *ab* plane. As shown in Table 1, the Bi_2_Te_3−x_Se_x_ bulk samples further prepared by SPS on different conditions all show relative densities higher than 98.5%. However, the compositions deviate a little to the lack of tellurium and selenium, as measured by ICPOES.

**Table 1:** Relative densities, orientation factors and compositions of the Bi_2_Te_3_ compound synthesized by FS and the Bi_2_Te_3−x_Se_x_ bulk samples further prepared by SPS on different conditions.

Samples	Sintering temperature (K)	Pressure (MPa)	Holding time (min)	Relative density (%)	Orientation factor	Actual composition
Bi_2_Te_3_(FS)	–	–	–	70.4 ± 0.1	0.013	Bi_2_Te_3.06_
Bi_2_Te_3_(SPS)	723	40	3	98.8 ± 0.1	0.079	Bi_2_Te_3.01_
Bi_2_Te_3_(SPS)	753	40	2	98.6 ± 0.1	0.083	Bi_2_Te_2.99_
Bi_2_Te_3_(SPS)	753	40	3	98.5 ± 0.1	0.095	Bi_2_Te_2.99_
Bi_2_Te_2.7_Se_0.3_(SPS)	753	40	3	98.8 ± 0.1	0.083	Bi_2_Te_2.69_Se_0.29_

**Figure 3:**
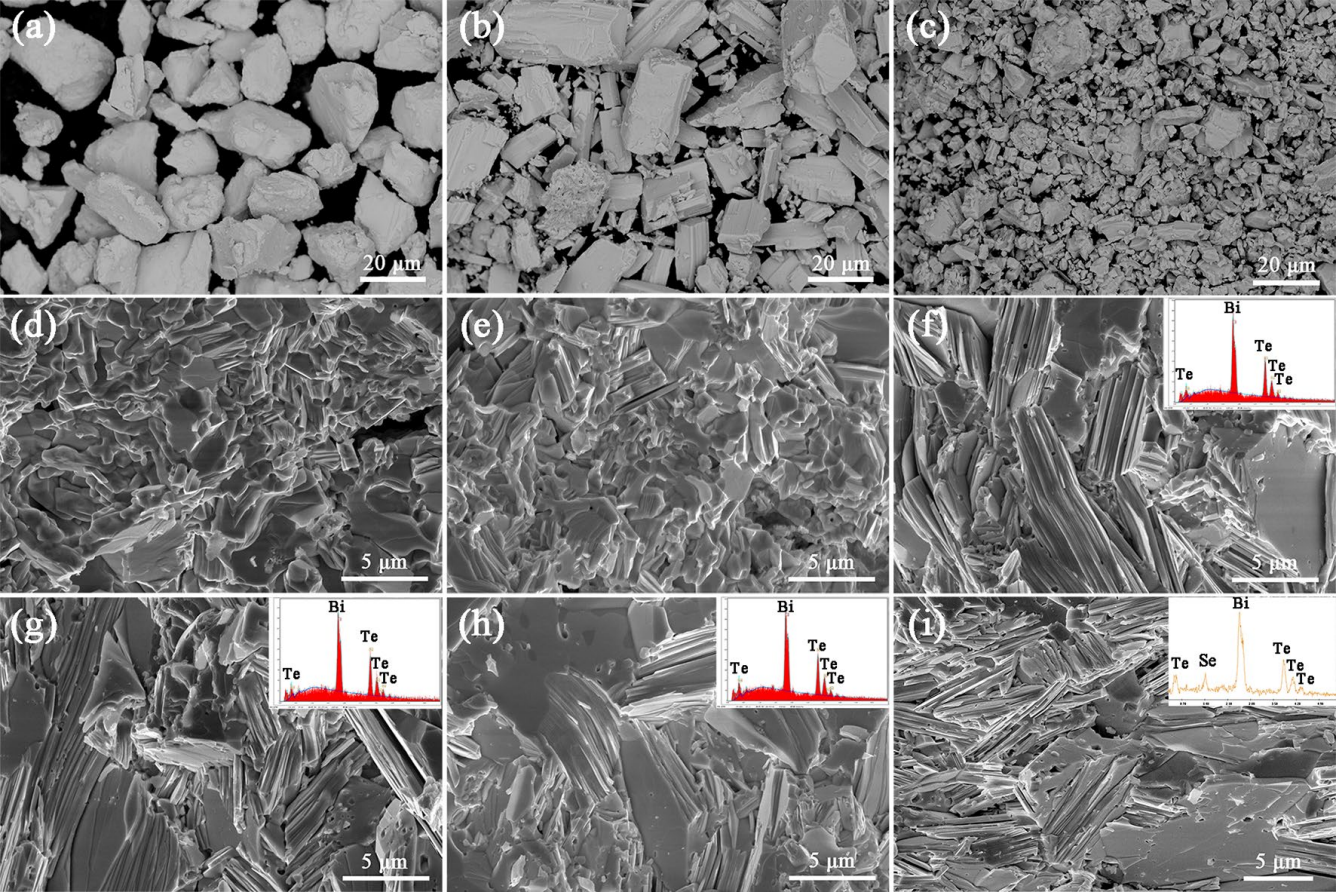
SEM images of (**a**) Bi, (**b**) Te and (**c**) Se raw powders. Cross-section SEM images of (**d**) Bi_2_Te_3_ compound synthesized by FS, (**e**) Bi_2_Te_2.7_Se_0.3_ compound synthesized by FS, (**f**) Bi_2_Te_3_ sample further prepared by SPS at 723 K for 3 min, (**g**) Bi_2_Te_3_ sample further prepared by SPS at 753 K for 2 min, (**h**) Bi_2_Te_3_ sample further prepared by SPS at 753 K for 3 min, (**i**) Bi_2_Te_2.7_Se_0.3_ sample further prepared by SPS at 753 K for 3 min. The insets show the EDS patterns.

The SEM images of Bi, Te and Se raw powders are shown in Fig. 3a–c, which have powder size of about 10–30 μm. For comparison, the Bi_2_Te_3_ and Bi_2_Te_2.7_Se_0.3_ compounds synthesized by FS have high crystallinity and compactness, as shown in Fig. 3d and e. Moreover, the size of the generated crystalline grain declines to about 1–3 μm, indicating that polycrystalline Bi_2_Te_3_ compound with much smaller grain size can be synthesized with micron Bi, Te and Se powders by FS. Thus, it shows great promise to synthesize Bi_2_Te_3_ based compounds with nanocrystalline by using nanoscale raw powders. The fracture morphologies of the Bi_2_Te_3_ based bulk materials subsequently prepared by SPS on different conditions are shown in Fig. 3f–i. The Bi_2_Te_3_ bulk samples prepared by SPS on different conditions are all highly dense with the typical layered structure. With the increase of sintering temperature and holding time in SPS process, it can be seen that the grain size becomes more homogeneous and the amount of nano cavity decreases because of more sufficient element diffusion and grain growth. Compared with the sample synthesized by FS, grain size increases to about 3–8 μm after SPS process. The grain structures of the Bi_2_Te_3_ bulk samples prepared by SPS on different conditions show no obvious preferred orientation, which are consistent with the results of XRD. The energy dispersive X-ray spectroscopy (EDS) patterns illustrate that the products only contain Bi, Te and Se elements, as shown in the insets in Fig. 3f–i. Due to the high-efficiency, low energy consumption and convenient operation process, FS combining with SPS can be considered as a promising method for the large-scale fabrication of Bi_2_Te_3_ based polycrystalline thermoelectric materials.

### Thermoelectric transport properties

**Figure 4:**
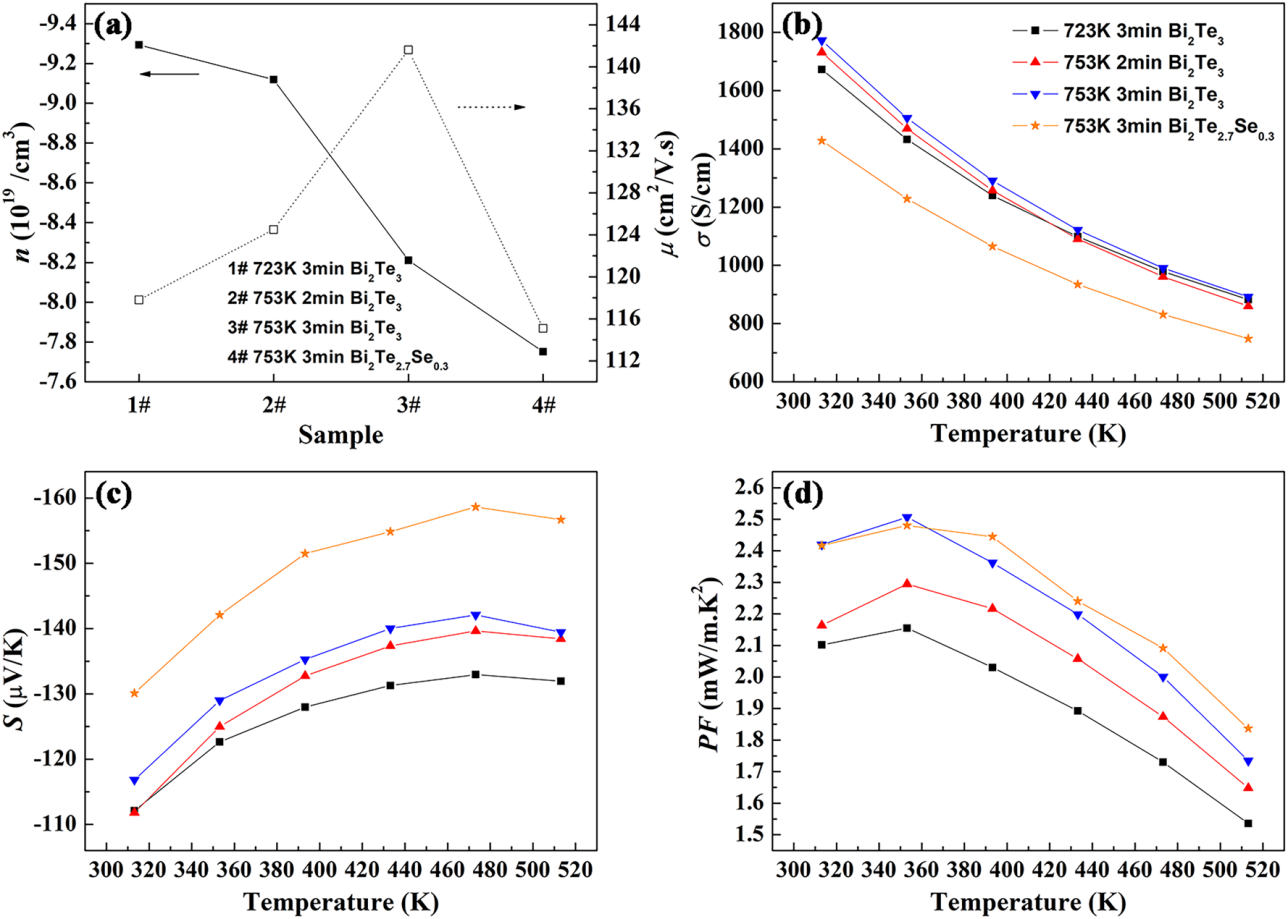
Electrical transport properties of the bulk Bi_2_Te_3−x_Se_x_ samples prepared by SPS on different conditions. (**a**) Carrier concentration *n* and mobility *μ* at 313 K, (**b**–**d**) Temperature dependence of electrical conductivity *σ*, Seebeck coefficient *S* and power factor *PF*.

To validate the effects of sintering temperature and holding time in SPS process on electrical transport properties of the samples, numerical values for carrier concentration (*n*) and mobility (*μ*) derived from Hall effect measurements at 313 K perpendicular to the SPS pressing direction are shown in Fig. 4a. All samples have negative values of carrier concentration, manifesting the dominance of electron in the transport process. With the increase of sintering temperature and holding time, carrier concentration decreases and carrier mobility increases. When sintering temperature and holding time increase, more power is supplied to the sample, which would lead to the volatilization of Te, as evidenced by the composition analysis in Table 1. Bi atom will enter into the Te vacancy because of the small difference in electronegativity between Bi and Te and more antisite defect Bi_Te_ will be produced. That means more hole could be generated to neutralize electron in these n-type samples, resulting in decreased carrier concentration. Absolute values of carrier concentration are around 8 × 10^19^–9 × 10^19^/cm^3^ for these samples, which is more than an order of magnitude higher than that of conventional zone melted sample^33^. Generally, the ion defects generated in sample under high electric field are tend to ionize to electron and uncharged lattice defect^34^. Therefore, the large carrier concentration probably arises from the effect of electric field in FS process. Compared with Bi_2_Te_3_ samples, Bi_2_Te_2.7_Se_0.3_ sample shows lower carrier concentration. For n-type Bi_2_Te_3_ based compounds, antisite defect Te_Bi_ is the dominant point defect which determines the carrier concentration. Although the equivalent dopant of Se is neutral, it may affect the formation of intrinsic point defect Te_Bi_ and thus the carrier concentration^35–37^. On the other hand, carrier mobility increases obviously from about 118 cm^2^/V s to 141 cm^2^/V s when the sintering temperature increases from 723 to 753 K for 3 min. The higher carrier mobility should be brought by the increased crystallinity of the product and the further eliminated microstructure defect at higher sintering temperature and longer holding time. After selenium doping, the carrier mobility decreases to 115 cm^2^/V s mainly due to the enhanced alloy scattering.

Thermoelectric properties of the bulk Bi_2_Te_3−x_Se_x_ samples prepared by SPS on different conditions were measured along the direction perpendicular to the SPS pressing direction. Figure 4b presents the change of electrical conductivity with measuring temperature. In whole measuring temperature range, the electrical conductivities decrease monotonically with the increasing measuring temperature for all samples, indicating a degenerate semiconductor feature. With the increase of sintering temperature and holding time in SPS process, the electrical conductivity increases slightly. Electrical conductivity *σ* is determined by carrier concentration and mobility as described by the relationship *σ* = *neμ*, where *n* is carrier concentration, *μ* is carrier mobility, and *e* is the electronic charge. Although carrier concentration decreases with the increase of sintering temperature and holding time because of the increased volatilization of Te and the resulted more antisite defect Bi_Te_, carrier mobility is obviously elevated due to more sufficient element diffusion and grain growth, as evidenced in Fig. 4a. Therefore, the enhanced carrier mobility counteracts the effect of decreased carrier concentration and results in a higher electrical conductivity. The electrical conductivity at room temperature of the sample prepared at 753 K for 3 min is approximate 1800 S/cm, which is even higher than that of the specimen prepared by hot pressing followed with hot deformation^38^. Because carrier concentration and mobility both decrease after partial substitution of selenium for tellurium due to the reduction of the number of intrinsic point defect Te_Bi_ and enhancement of alloy scattering as discussed above, Bi_2_Te_2.7_Se_0.3_ sample shows lower electrical conductivity than Bi_2_Te_3_ samples.

Figure 4c shows the temperature dependence of Seebeck coefficient for the bulk Bi_2_Te_3−x_Se_x_ samples prepared by SPS on different conditions. Seebeck coeffcients of the samples all exhibit negative values over the entire measuring temperature range, indicating as n-type semiconductors, which is consistent with the results of Hall effect measurements. With the increasing sintering temperature and holding time in SPS process, the absolute value of Seebeck coeffcient increases evidently. The Seebeck coeffcient of Bi_2_Te_3_ prepared at 723 K for 3 min is about – 132 μV/K at 473 K and the value is promoted to – 142 μV/K at 473 K for the sample prepared at 753 K for 3 min. According to Boltzmann transport theory, Seebeck coeffcient is inversely proportional to carrier concentration for solid thermoelectric materials. Therefore, the enhanced Seebeck coeffcient is mainly due to the decreased carrier concentration, as shown in Fig. 4a. Furthermore, the Bi_2_Te_2.7_Se_0.3_ sample shows much higher Seebeck coeffcients than Bi_2_Te_3_ samples. The promoted Seebeck coeffcient is also due to the lower carrier concentration after partial substitution of selenium and the resulted reduction of intrinsic point defect Te_Bi_. Seebeck coeffcients of the samples increase with increasing measuring temperature to about 480 K and then decrease slowly, showing a distinct temperature dependence from zone melted sample which shows monotonical downtrend^39^. The increase of Seebeck coeffcient in a wide temperature range is beneficial for the sustainability of high power factor.

Figure 4d shows the temperature dependence of power factor for the bulk Bi_2_Te_3−x_Se_x_ samples prepared by SPS on different conditions, which is calculated from electrical conductivity and Seebeck coefficient. With the increase of sintering temperature and holding time in SPS process, power factor is much enhanced owing to the increase in electrical conductivity and Seebeck coeffcient. Power factor of the sample prepared at 753 K for 3 min reaches to 2.5 mW/m K^2^ at 353 K, almost being 16% improvement compared with the sample prepared at 723 K for 2 min. Due to the high electrical conductivity and modest Seebeck coefficient of the sample, this value of power factor is even higher than that of the sample prepared by high-energy ball milling combining with SPS using commercial Bi and Te powders^40^. Furthermore, the value of power factor remains above 1.7 mW/m K^2^ in the whole measuring temperature range of 200 K, showing good stability with temperature. Although electrical conductivity decreases after partial substitution of selenium for tellurium, Bi_2_Te_2.7_Se_0.3_ shows even higher power factor than Bi_2_Te_3_ due to the enhanced Seebeck coefficient.

**Figure 5:**
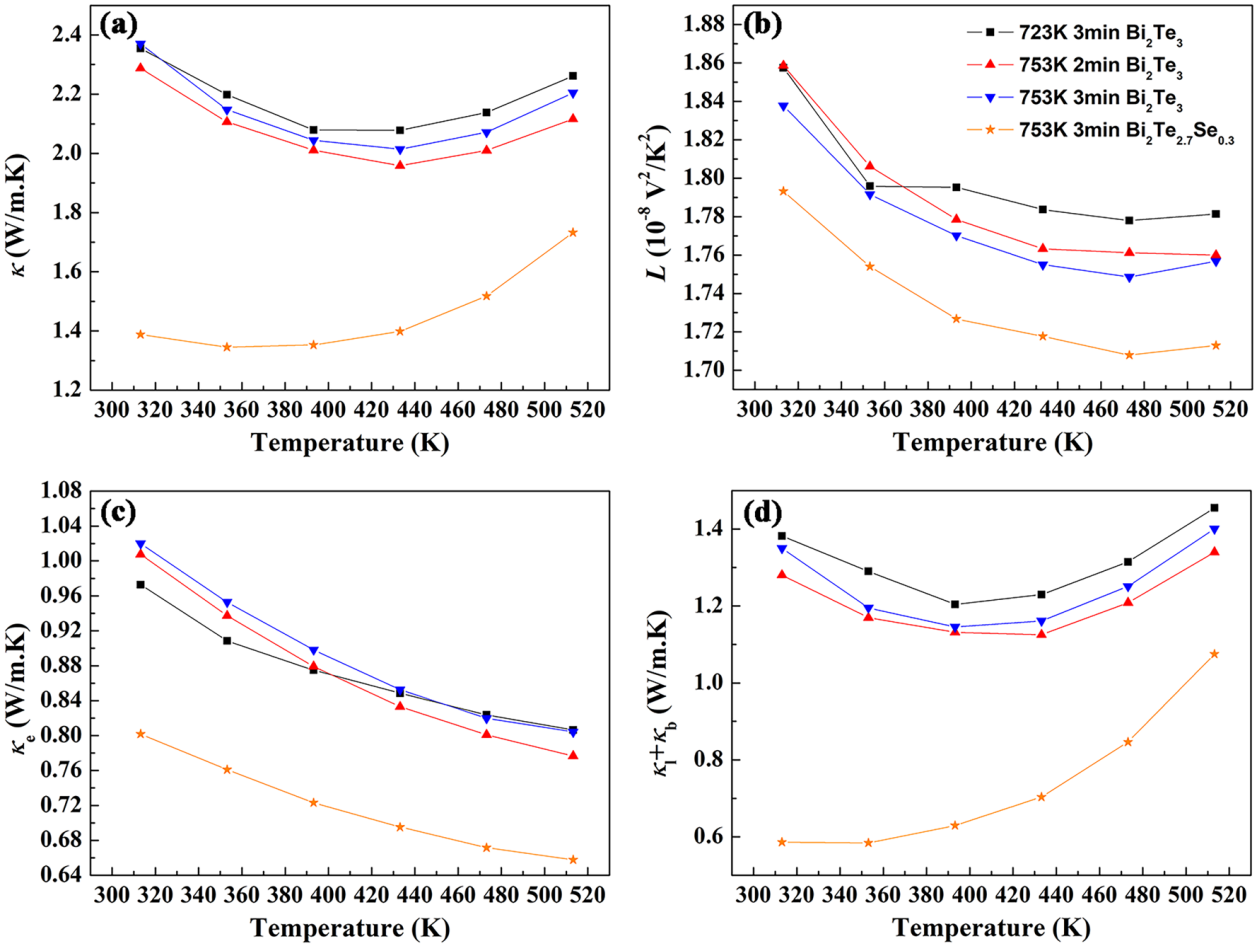
Thermal transport properties of the bulk Bi_2_Te_3−x_Se_x_ samples prepared by SPS on different conditions. Temperature dependence of (**a**) total thermal conductivity *κ*, (**b**) Lorentz number *L*, (**c**) electronic thermal conductivity *κ*_e_ and (**d**) lattice thermal conductivity *κ*_l_ plus bipolar thermal conductivity *κ*_b_.

The temperature dependence of thermal conductivity for the bulk Bi_2_Te_3−x_Se_x_ samples prepared by SPS on different conditions is shown in Fig. 5. The total thermal conductivity *κ* as illustrated in Fig. 5a decreases with increasing sintering temperature in SPS process over the entire measuring temperature range. However, with the increase of holding time, the thermal conductivity increases. Therefore, the sample prepared at 753 K for 2 min shows the lowest value of thermal conductivity. The electronic thermal conductivity *κ*_e_ can be estimated by the Wiedemann−Franz law *κ*_e_ = *LσT*, where *L* is the Lorentz number, *σ* is the measured electrical conductivity, and *T* is the absolute temperature. Based on the single parabolic band (SPB) model under relaxation time approximation, temperature dependent Lorenz number *L* can be calculated by the following equations:

3 $$L = \left( {\frac{{k_{B} }}{e}} \right)^{2} \left\{ {\frac{{\left( {\lambda + \frac{7}{2}} \right)F_{{r + \frac{5}{2}}} \left( \eta \right)}}{{\left( {\lambda + \frac{3}{2}} \right)F_{{r + \frac{1}{2}}} \left( \eta \right)}} - \left[ {\frac{{\left( {\lambda + \frac{5}{2}} \right)F_{{r + \frac{3}{2}}} \left( \eta \right)}}{{\left( {\lambda + \frac{3}{2}} \right)F_{{r + \frac{1}{2}}} \left( \eta \right)}}} \right]^{2} } \right\}$$

4 $$F_{n} \left( \eta \right) = \int_{0}^{\infty } {\frac{{x^{n} }}{{1 + e^{x - \eta } }}} dx$$

where *k*_B_ is the Boltzmann constant, *e* is the electron charge, *λ* is the scattering parameter (*λ* = − 1/2 for acoustic phonon scattering) and η is the reduced Fermi energy^8^. The determination of η is based on the measured Seebeck coefficient *S*, which is expressed as

5 $$S = \pm \frac{{k_{B} }}{e}\left[ {\frac{{\left( {\lambda + \frac{5}{2}} \right)F_{{\lambda + \frac{3}{2}}} \left( \eta \right)}}{{\left( {\lambda + \frac{3}{2}} \right)F_{{\lambda + \frac{1}{2}}} \left( \eta \right)}} - \eta } \right]$$

As shown in Fig. 5c, the electronic thermal conductivities *κ*_e_ decrease monotonically with the increasing measuring temperature for all samples, which show the same trend as that of electrical conductivity. The temperature dependence of *κ*_l_ + *κ*_b_ is illustrated in Fig. 5d. Compared with the value of *κ*_e_, *κ*_l_ + *κ*_b_ has larger contribution to the total thermal conductivity for Bi_2_Te_3_ samples. Because Seebeck coefficients all have the maximum value at around 480 K as discussed above, the intrinsic excitation should not occur below 400 K for all samples. That means bipolar thermal conductivity *κ*_b_ can be ignored and lattice thermal conductivity *κ*_l_ occupies the largest part of total thermal conductivity below 400 K. Although carrier concentrations and electrical conductivites are relatively high for these Bi_2_Te_3_ samples, phonon is still the predominant carrier for heat transport below 400 K. It is worth noting that *κ*_e_ and *κ*_l_ + *κ*_b_ are both decreased by partially substituting selenium for tellurium. Especially, the lattice thermal conductivity *κ*_l_ is lower than the electronic thermal conductivity *κ*_e_ for Bi_2_Te_2.7_Se_0.3_ below 400 K, indicating that phonon can been scattered greatly by alloying selenium due to the mass and size difference between Te and Se. As the Debye temperature of Bi_2_Te_3−x_Se_x_ is as low as 156 K, the main mechanism of phonon scattering is based on Umklapp process at near room temperature and lattice thermal conductivity *κ*_l_ would be inversely related to temperature. The total thermal conductivities all decrease and then increase with the increase of measuring temperature, as shown in Fig. 5a. Because electronic and lattice thermal conductivities both decrease monotonically with the increasing measuring temperature, bipolar thermal conductivity becomes a major contributor to the total thermal conductivity at high temperature.

**Figure 6:**
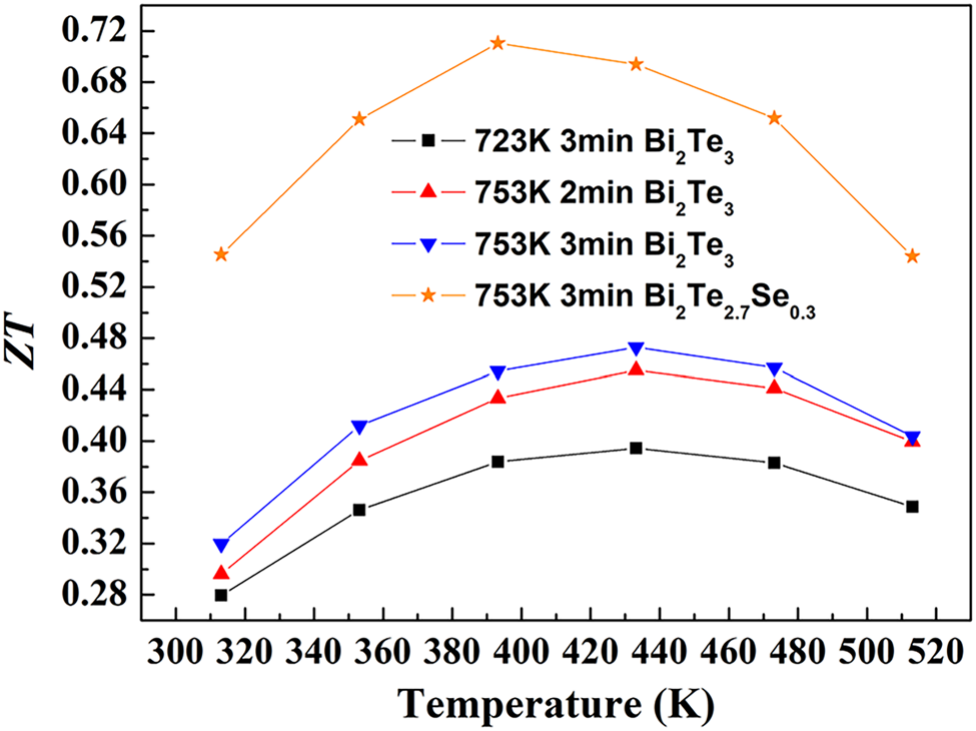
Temperature dependence of *ZT* values of the bulk Bi_2_Te_3−x_Se_x_ samples prepared by SPS on different conditions.

Figure 6 shows the temperature dependence of dimensionless figure of merit (*ZT*) for the bulk Bi_2_Te_3−x_Se_x_ samples prepared by SPS on different conditions. *ZT* values of the Bi_2_Te_3_ samples increase with temperature initially and then decrease at around 430 K. As dicussed above, the enhancement of power factor and suppression of thermal conductivity have been realized simultaneously by properly increasing the sintering temperature and holding time in SPS process. Therefore, the Bi_2_Te_3_ sample prepared at 753 K for 3 min shows 20% higher *ZT* value than that of the sample prepared at 723 K for 3 min. After partial substitution of selenium for tellurium, Bi_2_Te_2.7_Se_0.3_ shows a *ZT* value of 0.71 at 393 K. Although smaller than that of state-of-the-art, this value is actually comparable to those obtained in Bi_2_Te_3_ based nanocomposite bulk materials prepared by SPS and some other reference data^41–44^.

## Conclusions

In summary, FS method has been successfully employed to synthesize highly crystalline Bi_2_Te_3_ based compounds with small grain size in 10 s at room temperature using commercial Bi, Te and Se powders with much larger size. The instantaneously generated local Joule heat at grain boundary by passing current through sample is regarded as the main cause for the rapid completion of chemical reaction and crystallization. This rapid synthesis method at room temperature can significantly restrain grain growth and avoid tellurium volatilization. By combining FS synthesis method with SPS technique, Bi_2_Te_3_ based bulk materials with high relative density and thermoelectric performance can be fabricated in 10 min. Compared with common zone melting or powder metallurgy methods taking several hours by complex operation, this method is time-saving and low cost. In SPS process, the enhancement of electrical transport property and suppression of thermal transport property can be simultaneously obtained by suitably prolonging sintering temperature and holding time. Compared with the sample prepared at 723 K for 3 min, the sample prepared at 753 K for 3 min shows 20% higher *ZT* value. The results demonstrate that FS combining with SPS is a promising method to prepare bismuth telluride based thermoelectric materials efficiently and economically.

## Materials and methods

### Preparation of Bi_2_Te_3_ based bulk materials

Commercial Bi (99.99%, Macklin Inc.), Te (99.99%, Macklin Inc.) and Se (99.99%, Macklin Inc.) powders under 200 mesh were used for synthesis without any further purification. The raw powders were weighed according to the stoichiometric composition of Bi_2_Te_3−x_Se_x_ and then ground uniformly in an agate mortar. The mixture was loaded into a Φ13.2 mm graphite mold, the inner wall of which was insulated by a piece of mica paper to ensure that current only passed through the mixture rather than the graphite mold. Two Φ12.8 mm stainless-steel punches were used to press the mixed powder under 1.5 MPa by tablet press. Then the pressure was released, and the upper and lower punches were connected to a direct current power supply (PPS 2416, Lanyi, China). Voltage of 10 V and current limit of 32.15 A were preset. The sample was maintained at the current limit for 10 s at room temperature, before the power supply was turned off. The obtained product was further ground to powder and then sintered to compact bulk materials under a pressure of 40 MPa at 723 ~ 753 K for 2 ~ 3 min by SPS. The heating rate was set to be 80 K/min and the vacuum degree of cavity was under 6 Pa in SPS process. The obtained disk-shaped samples with diameter of 12.8 mm and height of 11.5 mm were cut along the direction perpendicular to the pressuring direction for thermoelectric performance measurement.

### Characterization

Phase compositions and crystallographic structures of the samples were identified by X-ray diffraction (XRD, Rigaku D/MAX 2200 PC) measurements using Cu Kα radiation (λ = 0.154056 nm). Morphologies and compositions of the raw powders and prepared products were investigated by field emission scanning electronic microscopy (FE-SEM, FEI Sirion 200) equiped with energy-dispersive spectroscope (EDS). Accurate elemental content analyses were carried out on a inductively coupled plasma optical emission spectrometer (ICPOES, Agilent ICPOES730). Electrical conductivities (*σ*) and Seebeck coefficients (*S*) were simultaneously measured using a commercial instrument (Cryoall CTA-3S) under a helium atmosphere. Hall coefficients (*R*_H_) were measured at near room temperature by van der Pauw method on a commercial Hall effect measurement system (Nanometrics HL5500PC). The values of carrier concentrations (*n*) were calculated via *n* = 1/(*eR*_H_) and the in-plane carrier mobilities (*μ*) were obtained through *μ* = *σR*_H_. The values of thermal conductivitie (*κ*) were calculated according to the relation *κ* = *DC*_p_*ρ*, where the thermal diffusivities (*D*) were determined by the laser flash method (Netzsch LFA475), specific heats (*C*_p_) were measured on a differential scanning calorimeter (TA DSC25), and mass densities (*ρ*) were determined by the method of Archimedes. To eliminate the effect of preference orientation on thermoelectric properties, the measuring of electrical and thermal properties are both along the direction perpendicular to the SPS pressing direction.

## Data Availability

The datasets generated during and/or analysed during the current study are available from the corresponding author on reasonable request.
